# *Fusobacterium necrophorum* Promotes Apoptosis and Inflammatory Cytokine Production Through the Activation of NF-κB and Death Receptor Signaling Pathways

**DOI:** 10.3389/fcimb.2022.827750

**Published:** 2022-06-14

**Authors:** Feng-Feng Wang, Peng-Yu Zhao, Xian-Jing He, Kai Jiang, Tian-Shuo Wang, Jia-Wei Xiao, Dong-Bo Sun, Dong-Hua Guo

**Affiliations:** Heilongjiang Provincial Key Laboratory of Prevention and Control of Bovine Diseases, College of Animal Science and Veterinary Medicine, Heilongjiang Bayi Agricultural University, Daqing, China

**Keywords:** Apoptosis, *Fusobacterium necrophorum*, inflammatory, RAW246.7 cells, sheep neutrophil

## Abstract

*Fusobacterium necrophorum* can cause liver abscess, foot rot in ruminants, and Lemire syndrome in humans, Also, its virulence factors can induce the apoptosis of macrophages and neutrophils. However, the detailed mechanism has not been fully clarified. This study investigated the mechanisms of apoptosis and inflammatory factor production in *F. necrophorum*–induced neutrophils and macrophages (RAW246.7). After infection of macrophages with *F. necrophorum*, 5-ethynyl-2’-deoxyuridine labeling assays indicated that *F. necrophorum* inhibited macrophage proliferation in a time- and dose-dependent manner. Hoechst staining and DNA ladder assays showed significant condensation of the nucleus and fragmentation of genomic DNA in *F. necrophorum*–infected macrophages, Annexin V (FITC) and propidium iodide (PI) assay confirmed the emergence of apoptosis in the macrophages and sheep neutrophils with *F. necrophorum* compared with the control. The group with significant apoptosis was subjected to RNA sequencing (RNA-Seq), and the sequencing results revealed 2581 up– and 2907 downregulated genes. Gene Ontology and Kyoto Encyclopedia of Genes and Genomes analysis of the differentially expressed genes showed that *F. necrophorum* drove apoptosis and production of inflammatory factors by activating genes related to the Nuclear Factor-κB (NF-κB) and death receptor pathways. Meanwhile, quantitative reverse transcription PCR and Western blot validation results were consistent with the results of transcriptome sequencing analysis. In conclusion, *F. necrophorum* induced apoptosis and production of pro-inflammatory factors through the NF-κB and death receptor signaling pathway, providing a theoretical basis for further mechanistic studies on the prevention and control of *F. necrophorum* disease treatment.

## Introduction

*Fusobacterium necrophorum* (*F. necrophorum*) belongs to the family Fusobacteriaceae, is a Gram-negative, rod-shaped, non-flagellated, strictly anaerobic bacterium that does not form spores and pods. It is mainly found in the digestive and genitourinary tracts of humans and animals and it is also common in nature. Various livestock, poultry, and wild animals are susceptible to *F. necrophorum* (Tan et al., 1996; Nagaraja et al., 2005). The species is classified into two subspecies based on the biological and biochemical characteristics, DNA base composition, and DNA–DNA homology level of the two biotypes of *F. necrophorum*: *subsp. necrophorum* and *subsp. funduliforme* (Shinjo et al., 1991). The *subsp. funduliforme* mainly infects humans and causes *Lemierre′s* syndrome. The initial symptoms of this disease are pharyngitis and secondary jugular vein septic thrombophlebitis (Lazar et al., 2021), but occasionally liver abscess (Radovanovic et al., 2020) and calf fasciitis. The *subsp. necrophorum* is related to necrotizing and suppurative infections in animals, such as liver abscess, foot rot, and endometritis (Langworth, 1977).

Several virulence factors have been reported contributing to the pathogenesis of *F. necrophorum* infection, including leukotoxin (Lkt), lipopolysaccharide (LPS), hemolysin, hemagglutinin, capsule, adhesins, platelet aggregation factor, dermonecrotic toxin, and several extracellular enzymes such as proteases and deoxyribonucleases (Tadepalli et al., 2008). In the presence of hemolysin, *F. necrophorum* consumes oxygen at the site of infection, creating an anaerobic environment for its growth and reproduction, which can exacerbate its condition, and enhance its colonization and invasion in different infected areas through an outer membrane protein (Amoako et al., 1997; Kumar et al., 2013). In contrast, endotoxin infection causes an increase in the numbers of neutrophils and macrophages (Garcia et al., 2000). However, cytotoxins could induce neutrophil apoptosis at a low concentration, but necrosis at a high concentration, and 90% of rabbit peritoneal macrophages were damaged to varying degrees within 6 h (Fales et al., 1977; Narayanan et al., 2002). *F. necrophorum* infection has been shown to promote high expression of the inflammatory cytokines TNF-α (tumor necrosis factor-α) and IL-1β (interleukin-1β) in hoof tissue (Davenport et al., 2014). However, apoptosis and inflammatory factor production mechanisms in *F. necrophorum*−induced neutrophils and macrophages have not been elucidated.

Inflammation and apoptosis are the first steps of defense by neutrophils and macrophages against the invasion of pathogenic microorganisms into an organism. Necrotizing and septic diseases caused by necrotizing bacilli are inseparably linked to the damage of neutrophils and macrophages in the organism. Although some studies have shown that *F. necrophorum* can induce the apoptosis of neutrophils and macrophages, the molecular mechanism of their regulation is not yet clear. Therefore, in this study, the effect of *F. necrophorum* on cell proliferation was first detected by the 5-ethynyl-2’-deoxyuridine (EdU) method. Then, the influence of *F. necrophorum* on macrophage and neutrophil apoptosis was further detected by Hoechst staining, DNA ladder assays, and flow cytometry. The effect of *F. necrophorum* on neutrophil gene expression was analyzed using transcriptomics. Finally, the mechanism of *F. necrophorum*−induced apoptosis and inflammatory factor production was verified at the gene and protein levels, respectively. So, it is inevitable to investigate the molecular mechanism of *F. necrophorum* induced cell damage. This study also provided the basis for the pathogenic mechanism between *F. necrophorum* and hosts.

## Materials and Methods

### Cells and Culture Conditions

Sheep neutrophils were extracted from healthy sheep blood using a sheep peripheral blood neutrophil extraction kit (P4150, Solarbio, Beijing, China); macrophages (RAW264.7) stored in Laboratory of Pathology, Heilongjiang Bayi Agricultural University were cultured in Dulbecco’s modified Eagle′s medium (DMEM) (Sigma, Shanghai, China) supplemented with 10% fetal bovine serum (FBS) (Clark Bioscience, Shanghai, China), 100 U penicillin mL^-1^, and 100 mg streptomycin mL^-1^ at 37°C with 5% CO_2_. This study was approved by the Animal Health, Animal Care, and Use Committee of the Heilongjiang Bayi Agricultural University.

### Bacterial Strain and Culture Conditions

*Fusobacterium necrophorum* subsp*. necrophorum* (*F. necrophorum*) strain was purchased from the American Type Culture Collection (ATCC 25286, VA, USA) and cultured in brain heart infusion (BHI) (Hopebiol, Qingdao, China) liquid or solid media at 37°C in the anaerobic incubator with 85% CO_2_, 10% H_2_, and 5% N_2_. The bacterial growth was monitored by measuring optical density at 600 nm (OD = 600), and the logarithmic growth phase was selected in experiments.

### Effects of *F. necrophorum* on Macrophage Proliferation

The macrophages were seeded in six-well (2 × 10^5^ cells/well) plates in a DMEM growth medium with 10% FBS. They were co-cultured with *F. necrophorum* under multiple infections (MOI) (*F. necrophorum*: 50:1, 100:1, 200:1, 500:1 and 1000:1 cells) for 2, 4 and 6 hours at 37°C, 5% CO_2_ incubator in the closed-culture, and the remaining untreated cells were used as control. The 5-ethynyl- 2′-deoxyuridine labeling assay was used to evaluate the cell proliferation rate according to the instructions of the BeyoClick EdU Cell Proliferation Kit with Alexa Fluor 488 (C0071L, Beyotime, Nanjing, China). The experiment was divided into six groups, and each group was repeated three times.

### Apoptotic Effect of *F. necrophorum* on Neutrophils and Macrophages

Hoechst 33258 staining: A Hoechst 33258 cell apoptosis staining kit (C1018, Beyotime, Nanjing, China) was used to confirm the morphological changes in the nuclei. The macrophages were seeded onto sterile glass coverslips placed in six-well plates and treated with or without *F. necrophorum* for 2, 4 and 6 h. The cells were fixed, washed three times with phosphate-buffered saline (PBS), and stained with Hoechst 33258 staining solution for 5 min at room temperature. The slides were evaluated, and the images were captured using an EVOS M5000 imaging system (Thermo Fisher Scientific, WA, USA). Apoptotic cells were defined by the condensation of nuclear chromatin or fragmentation to the nuclear membrane.

DNA fragmentation: The macrophages were seeded in six-well plates (2 × 10^5^ cells/well) and stimulated with different densities of *F. necrophorum* for 2, 4 and 6 h. Afterward, the collected cells were washed with PBS. The fragmented DNA was isolated with a DNA extraction kit (C0008, Beyotime, Nanjing, China) following the manufacturer’s protocols. The eluants containing DNA pellets were electrophoresed on a 1.5% agarose gel at 80 V for 1.5 h. The gel was examined and photographed using an ultraviolet gel documentation system.

Flow cytometry analysis of apoptosis: The cell apoptosis was analyzed using an Annexin V-FITC/PI kit (CA1020, Solarbio, Beijing, China). Sheep neutrophils and macrophages were seeded in six-well plates (2 × 10^5^ cells/well) and stimulated with different concentrations of *F. necrophorum* for 2 h, 4 h, and 6 h. Afterward, the collected cells were washed with PBS, and stained with Annexin V-FITC and PI for 15 min in the dark at room temperature. The cell apoptosis was analyzed using a FACSCalibur flow cytometer (BD Biosciences, NJ, USA). The results were expressed as the percentage of apoptotic cells among all the cells. The experiment was performed in triplicate.

### Transcriptomic Analysis of *F. necrophorum* Acting on Neutrophils

Sheep neutrophils were seeded on 6 well plates (2 × 10^5^ cells/well), stimulated with *F. necrophorum* with MOI of 100 for 4 hours, and the remaining untreated cells were used as the control. The experiment was divided into 2 groups, repeated 3 times in each group, with a total of 6 samples. A total of six samples were sequenced to analyze the gene expression at the whole genome level at Shanghai Biotree Tech (Shanghai, China) by RNA-seq. Total RNA was extracted, evaluated for quality, reverse transcribed into cDNA, and sequenced on the Illumina platform. After the quality control (QC) step (Agilent 2100 Bioanalyzer, CA, USA), the clean reading was mapped to the reference genome (oar_v3.1) through the hierarchical index of transcript splicing alignment (hisat2 v2.0.5), and the clean reading was mapped to the reference transcript using stringtie (1.3.3b). Fragments per Kilobase Million (FPKM) was used to calculate the gene expression level of each sample, and the correlation between all samples was detected by Pearson correlation between samples. Based on the gene expression level, the differentially expressed genes (DEGs) between the control group and the *F. necrophorum*–infected group were detected by DESeq2 (1.16.1) algorithms. The changes in the absolute logarithm base of DEG ≥1 and adjusted *P* value (%) <5% were detected. Gene Ontology (GO) was used to screen and annotate DEGs. ClusterProfile (3.4.4) software was used to generate GO function classification files. The pathway enrichment analysis of DEGs was carried out based on the Kyoto Encyclopedia of Genes and Genomes (KEGG) database. The Pathview website was used to analyze the signal pathway activation after stimulation by *F. necrophorum*. RNA sequence data have been stored in the NCBI Sequence Read Archive database (SRA, https://www.ncbi.nlm.nih.gov/sra/)Accessible *via* sra series accession number (PRJNA783192).

### RNA Isolation and Real-Time PCR

Sheep neutrophils and macrophages were seeded in six-well plates and cultured with *F. necrophorum*. The cells were collected at three different time points (2, 4 and 6 h), and total RNA was extracted using TRIzol (Ambion, TX, USA). The mRNA concentration was determined using a Titertek Berthold Colibri ultramicro spectrophotometer (Titertek-Berthold, Pforzheim, Germany). One microgram of mRNA was reverse transcribed into cDNA using a PrimeScript RT reagent Kit with a gDNA Eraser (TaKaRa, Beijing, China). RT-qPCR was performed with a TaKaRa TB Green Premix Ex Taq (Tli RNaseH Plus; TaKaRa, Beijing, China) on a light Cycler 96 Real-Time PCR System (Bio-Rad, Shanghai, China), β-Actin and GAPDH were used as housekeeping genes in neutrophils and macrophages, respectively. The reaction conditions were as follows: first, pre-denaturation at 95°C for 30 s, then denaturation at 95°C for 5 s, and annealing at 60°C for 30 s. The aforementioned steps involved 40 cycles at 95°C for 10 s. The dissolution curve was set as 65°C for 5 s, and 95°C for 5 s. The data were analyzed using the 2^-ΔΔCT^ method. The sequence of amplification primers is shown in **Tables 1**, **2**. Each test was repeated three times.

**Table 1:** Sheep neutrophils primer sequences.

Name	Forward Primer	Reverse Primer
IL-1β	GAAGAGCTGCACCCAACACCTG	CGACACTGCCTGCCTGAAGC
TNF-α	AACAGGCCTCTGGTTCAGACA	CCATGAGGGCATTGGCATAC
IL-6	TCAGTCCACTCGCTGTCTCC	TCTGCTTGGGGTGGTGTCAT
Bcl-2	☐GCCGAGTGAGCAGGAAGAC	GTTAGCCAGTGCTTGCTGAGA
Bax	CAGAGGCGGGGTTTCATCC	TCGGAAAACATTTCAGCCGC
Cytc	CAGAAGTGTGCCCAGTGCCATAC	GCCTGACCTGTCTTTCGTCCAAAC
Caspase-3	AGCCTTCATTCTTCGTGCCACAG	CGACTGAGCGACTGAACTGAACTG
JNK	GCTGTGTACATGTCGGCTTC	TGAGTGACCCTGTTTAGCCA
TRAF2	CCTTCGGAGAAGATGATGGGG	TTCCTTACGCACACCCCAAG
β-actin	CCACAGCCGAGCGGGAAATTG	AGGAGGACGACGCAGCAGTAG

**Table 2:** Macrophage primer sequences.

Name	Forward Primer	Reverse Primer
IL-6	CGGAGAGGAGACTTCACAGAG	ATTTCCACGATTTCCCAGAG
IL-1β	GCACTACAGGCTCCGAGATGAAC	TTGTCGTTGCTTGGTTCTCCTTGT
TNF-α	TACTGAACTTCGGGGTGATTGGTCC	CAGCCTTGTCCCTTGAAGAGAAC
Bax	CAGGATGCGTCCACCAAGAA	CAAAGTAGAAGAGGGCAACCAC
Bcl-2	CTACGAGTGGGATGCTGGAGA	CAGGCTGGAAGGAGAAGATGC
Caspase-3	GGCTGACTTCCTGTATGCTTACTCTAC	ACTCGAATTCCGTTGCCACCTTC
Caspase-8	ACCAAATGAAGAACAAACCTCG	CTTCATTTTTCGGAGTTGGGTT
Caspase-9	CGCCAAAATTGAAATTCAGACG	CGACAGGCCTGGATGATAAATA
Caspase-12	TGGCCCATGAATCACATCTAAT	TGGACAAAGCTTCAGTGTATCT
AIF	CATCATGATCATGCTGTTGTGA	TATCCACCAGACCAATAGCTTC
Cyto-c	GCAGGGTGCTAACTCAGTCC	CACTTAGGATCACCCCCAGC
Iκκα	GGTGGAGGCATGTTCGGTAG	CACTCTTGGCACAATCTTTAGGG
TRAF2	AACCTTTGAGAACATTGTCTGC	CCTCAATCTTGTCCTGGTCTAG
P65	CACCAAGGATCCACCTCACC	CTCTATAGGAACTATGGATACTGCG
PP65	ACATCAAGGACTCCAAAGCTTA	GTCCTGACATGTCAATCACAAC
JNK	GCTGTGTACATGTCGGCTTC	TGAGTGACCCTGTTTAGCCA
GAPDH	CGTGCCTGGAGAAACCTG	AGAGTGGGAGTTGCTGTTGAAGTCG

### Western Blot Assay

Macrophages with or without *F. necrophorum* stimulation were harvested with NP-40 lysis buffer (P0013F, Beyotime, Nanjing, China). The protein concentration was determined by bicinchoninic acid assay analysis, and proteins (30 μg/lane) were separated into 12% sodium dodecyl sulfate-polyacrylamide gel electrophoresis gels and transferred to polyvinylidene fluoride membranes (Eppendorf, Shanghai, China). The membrane was sealed with 5% skimmed milk at room temperature for 1 h and incubated with primary antibody at 4°C overnight. They were then incubated with horseradish peroxidase–conjugated secondary antibodies (SA00001-1 or SA00001-2, Proteintech, IL, USA; 1:10,000) for 1 h at room temperature. The protein bands on the film were covered with enhanced chemiluminescence reagent (Millipore, MA, USA) and scanned with an ultra-sensitive imager (Amersham Imager 600; Gen Healthcare Life Sciences, PA, USA). ImageJ 1.52a software (NIH, MD, USA) was used to quantify protein expression levels. The main antibodies and dilution rates were as follows: B-cell lymphoma-2 (BCL2) rabbit polyclonal antibody (12789-1-AP, Proteintech, IL, USA; 1:4000), caspase 3/p17/p19 rabbit polyclonal antibody (19677-1-AP, Proteintech, IL, USA; 1:1000), BCL2-Associated X (BAX)rabbit polyclonal antibody (50599-2-Ig, Proteintech, IL, USA; 1:1000), cytochrome c polyclonal antibody (ab133504, Abcam, Cambrdge, MA; 1:1000), rabbit anti-NF-κB p65 (10745-1-AP, Proteintech, IL, USA; 1:1000), and rabbit anti-phospho-NF-κB p65 (10159-2-AP, Proteintech, IL, USA; 1:1000), Toll-like receptor 4 (TLR4) mouse monoclonal antibody (66350-1-Ig, Proteintech, IL, USA; 1:2000), phospho- c-Jun N-terminal kinase (JNK, Tyr185) rabbit recombinant antibody (80024-1-RR, Proteintech, IL, USA; 1:2000), c-Jun N-terminal kinase (JNK) mouse monoclonal antibody (66210-1-Ig, Proteintech, IL, USA; 1:5000), rabbit anti-AIF antibody (ab32516, Abcam, Cambrdge, MA; 1:1000), caspase-8 mouse monoclonal antibody (sc-81656, SANTA, CA, USA; 1:200), caspase-9 mouse monoclonal antibody (sc-56076, SANTA, CA, USA; 1:200), TLR2 rabbit polyclonal antibody (17236-1-AP, Proteintech, IL, USA; 1:2000), TNF alpha polyclonal antibody (17590-1-AP, Proteintech, IL, USA; 1:1000), alpha tubulin monoclonal antibody (66031-1-Ig, Proteintech, IL, USA; 1:20,000), IL-6 monoclonal antibody (66146-1-Ig, Proteintech, IL, USA; 1:1000), TNF receptor-associated factors 2 (TRAF2) polyclonal antibody (26846-1-AP, Proteintech, IL, USA; 1:1000), IL-1 beta polyclonal antibody (16806-1-AP, Proteintech, IL, USA; 1:500), and Anti-IκB alpha antibody (ab32518, Abcam, Cambrdge, MA; 1:1000). The experiment was performed in triplicate.

### NF-κB Activation and Nuclear Translocation Assay

To detect *F. necrophorum*-induced nuclear translocation of NF-κB p65, RAW246.7 cells were seeded in coverslip containing 24-well plates at a density of 2 × l0^4^ cells/well and treated with or without *F. necrophorum* for 2 h to 6 h. Using the reagents provided in the kit, the cells were washed and fixed, and then incubated with blocking solution for 1 h at room temperature. Cells were incubated with rabbit anti-NF-κB p65 antibodies (SN371, Beyotime, Nanjing, China) overnight at 4°C. Following washing, the cells were further incubated with Cy3-conjugated secondary antibodies (SN371, Beyotime, Nanjing, China) for 1 h and 4’,6-diamidino-2-phenylindole (DAPI) for 5 min at room temperature. Images were captured by fluorescence microscopy (Leica, Wetzlar, Germany). The experiment was performed in triplicate and repeated three times.

### Enzyme-Linked Immunosorbent Assay

RAW246.7 cells were seeded in 6-well plates and treated with *F. necrophorum* at an MOI of 100: 1. Cell culture supernatants were collected and centrifuged at 1,000*g for 20 min at 4°C. The levels of secreted IL-6, IL-8, IL-1β, and TNF-α protein were measured by ELISA (USCN, Wuhan, China) according to the manufacturer’s instructions. The optical density values were measured by a microplate reader at 450 nm, The experiment was performed in triplicate and repeated three times.

### Statistical Analysis

Data were reported as the mean ± standard deviation (*n* = 3). The values were analyzed using the software GraphPad Prism 8. The two-way ANOVA command with Sidak’s multiple comparisons test were used to analyze the data differences among more than two groups. Compared with the control, *P* values less than 0.05 indicated a statistically significant difference (^∗^ *P* < 0.05, ^∗∗^ *P* < 0.01, and ^∗∗∗^ *P* < 0.001).

## Results

### *F. necrophorum* Promoted Macrophages to Undergo Apoptosis and Inflammation

**Figure 1:**
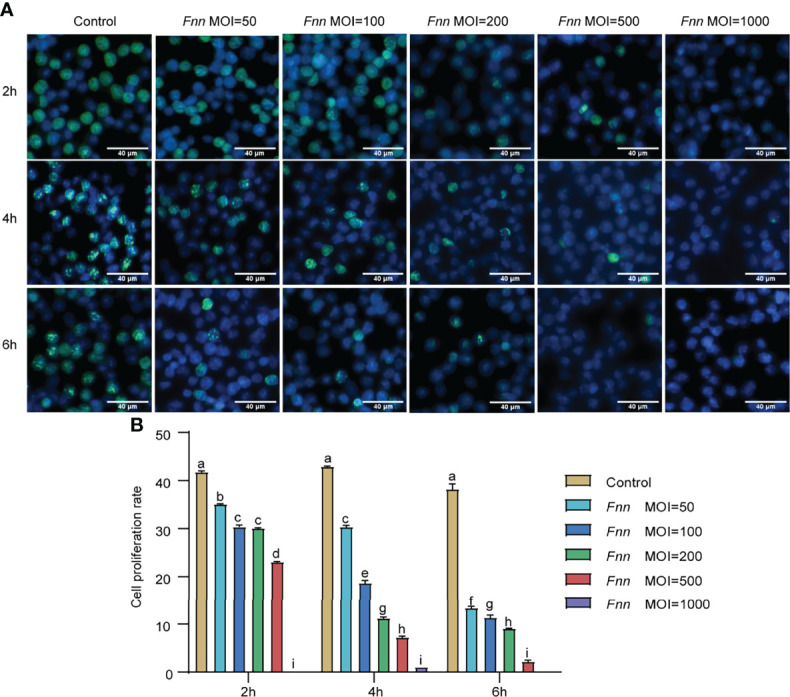
Effects of *F. necrophorum* on macrophages proliferation. **(A)** EdU assay of macrophages after *F. necrophorum* stimulation (MOIs of 0, 50, 100, 200, 500, and 1000) at 2, 4, and 6 h. Scale bar: 40 μm. **(B)** Cell proliferation rate of macrophages detected by EdU assay (n = 3), (No significance with the same letter, significant difference with different letters, *P* < 0.05).

The EdU-labeling assay was applied to quantify the cell proliferation rate after 2, 4, and 6 h after *F. necrophorum* stimulation at MOIs of 50, 100, 200, 500, and 1000. The results showed that the number of proliferating cells (yellow-green) decreased (**Figure 1A**). The statistical analysis indicated that *F. necrophorum* inhibited cell proliferation after macrophage infection with significant differences compared with the control group (**Figure 1B**). The EdU-labeling assay demonstrated that *F. necrophorum* inhibited macrophage proliferation in a time-and dose-dependent manner.

**Figure 2:**
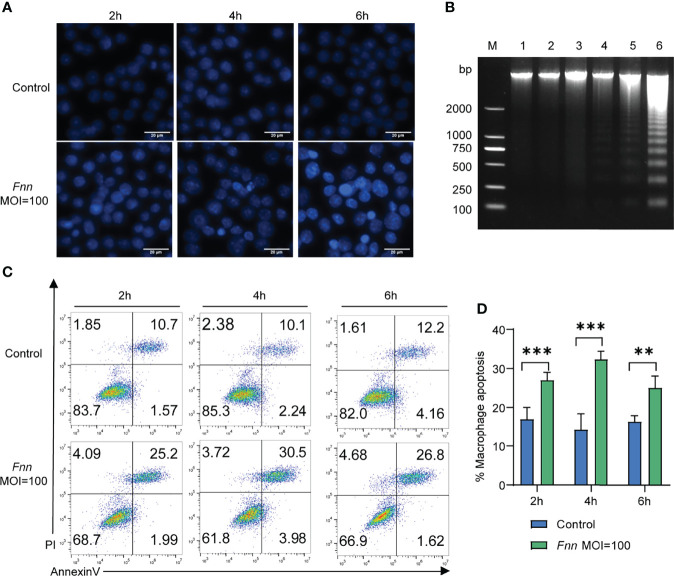
Apoptosis of macrophages cells induced by *F. necrophorum* (MOI=100). **(A)** Hoechst 33258 staining fluorescent display. **(B)** Effect of the *F. necrophorum* on chromosomal DNA fragmentation (Lane M: DNA marker 2000; Lane 1, 3, and 5: macrophages were cultured for 2, 4, and 6 h without *F. necrophorum*; Lane 2, 4, and 6: macrophages cultured for 2, 4 and 6 h incubated with *F. necrophorum*). **(C)**. Flow cytometry analysis of macrophages apoptosis with *F. necrophorum* stimulation (MOIs of 0 and 100) at 2, 4, and 6 h. **(D)**. Statistical analysis of macrophages apoptosis with *F. necrophorum* stimulation (MOIs of 0 and 100) at 2, 4, and 6 h. The histogram represents the mean ± SD (n = 3). **P<0.01, ***P<0.001.

Based on the inhibition of macrophage proliferation by *F. necrophorum*, *F. necrophorum*–infected macrophages with MOI of 100 were selected for apoptosis assay, as follows. Hoechst 33258 staining was used to analyze the apoptosis of macrophages infected with *F. necrophorum* (MOI = 100) for 2, 4, and 6 h under the fluorescence microscope. The morphology of living cells was round or oval standard blue, while the nucleus of apoptotic cells showed dense staining and was bright blue because of chromatin pyknosis. The fluorescence intensity of cells infected with *F. necrophorum* was detected at 2 h, 4 h and 6 h respectively in Hoechst staining experiment, and the results showed that the fluorescence intensity increased at 4h after infection, and was most obvious at 6 h (**Figure 2A**). DNA fragmentation showed an evident DNA gradient band in the infection group compared with the control group, which was time-dependent and the most apparent after 4 h (**Figure 2B**). The Annexin V-FITC/PI method was used to detect the *F. necrophorum*−infected apoptotic rate of macrophages. Flow cytometry showed that the apoptotic rate of macrophages in the control group was 12.27%, 12.25%, and 16.36%, respectively. The apoptotic rate of macrophages in the experimental group was 27.2%, 34.5%, and 28.4%, respectively (**Figure 2C**). The *F. necrophorum*−infected apoptotic rate of macrophages was significantly different at different time points (*P* < 0.01) (**Figure 2D**).

**Figure 3:**
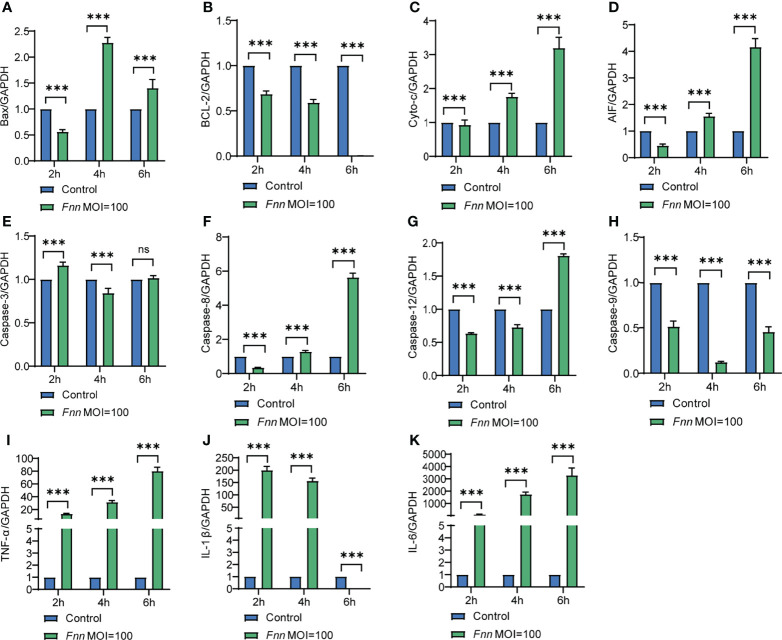
The effect of *F. necrophorum* on apoptosis and inflammatory factors of macrophages was detected by RT-qPCR. Bax **(A)**, Bcl-2 **(B)**, cyto-c **(C)**, AIF **(D)**, caspase-3 **(E)**, caspase-8 **(F)**, caspase-12 **(G)**, caspase-9 **(H)**, TNF- α **(I)**, IL-1 β **(J)** and IL-6 **(K)** gene expression with *F. necrophorum* stimulation (MOIs of 0 and 100) from 0 to 6 h (*n* = 3). ***P<0.001, ns, not significant.

The apoptosis-related genes Bcl-2, caspase-3, Bax, AIF, cyto-c, caspase-8, caspase-12, and caspase-9 and pro-inflammatory factors TNF-α, IL-1β, and IL-6 in *F. necrophorum*–infected macrophages were detected by RT-qPCR to further study the mechanism of *F. necrophorum* inhibiting macrophage proliferation and inducing apoptosis and the release of inflammatory cytokines. The results showed that the pro-apoptotic genes Bax (**Figure 3A**), cyto-c (**Figure 3C**), AIF (**Figure 3D**), caspase-3 (**Figure 3E**), caspase-8 (**Figure 3F**), caspase-12 (**Figure 3G**) and caspase-9 (**Figure 3H**) were upregulated and the anti-apoptotic gene Bcl-2 (**Figure 3B**) was downregulated. Pro-inflammatory factors TNF-α (**Figure 3I**), IL-1β (**Figure 3J**), and IL-6 (**Figure 3K**) showed an up-regulation trend (*P* < 0.01). The results showed that the infection of macrophages by *F. necrophorum* promoted the gene expression of apoptotic factors and inflammatory cytokines. Therefore, the inhibition of cell proliferation by *F. necrophorum* is closely related to the induction of apoptosis and inflammation.

**Figure 4:**
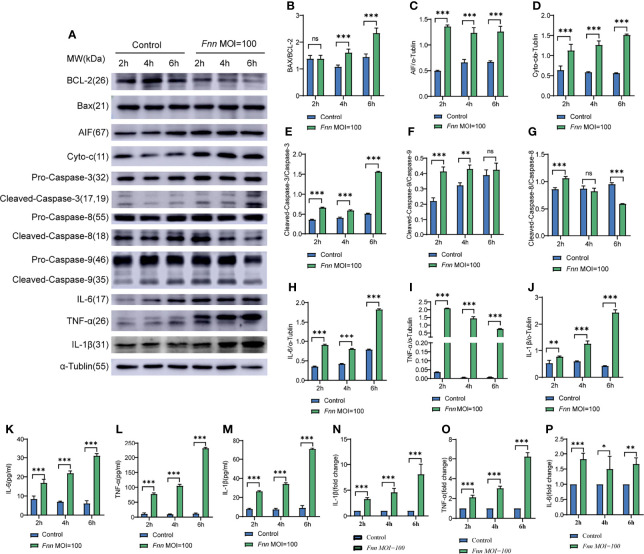
The effect of *F. necrophorum* on apoptosis and inflammatory factors of macrophages was detected by Western blot and ELISA. **(A)** The protein levels of Bcl-2, Bax, AIF, cyto-c, pro-caspase-3, cleaved-caspase-3, pro-caspase-9, cleaved-caspase-9, IL-6, TNF-α and IL-1β were detected by western blotting. The relative level of Bax/Bcl-2 **(B)**, AIF **(C)**, cyto-c **(D)**, cleaved-caspase-3/caspase-3 **(E)**, cleaved-caspase-9/caspase-9 **(F)**, cleaved-caspase-8/caspase-8 **(G)**, IL-6 **(H)**, TNF-α **(I)** and IL-1β **(J)** was detected. ELISA detection of the levels of IL-6 **(K)**, TNF-α **(L)** and IL-1β **(M)** in cells (*n* = 3). The ratio of IL-1β **(N)**, TNF-α **(O)** and IL-6 **(P)** between the Fnn MOI=100 group and the control group was detected by ELISA (n = 3). *P<0.05, **P<0.01, ***P<0.001, ns, not significant.

The apoptosis-related proteins Bcl-2, caspase-3, Bax, AIF, and cyto-c in *F. necrophorum*–infected cells were detected using Western blot analysis to investigate the effects of *F. necrophorum* infection on apoptosis, inflammatory response, and other change. The results are shown in **Figure 4A**. The protein expression ratio of the pro-apoptotic protein Bax to the inhibitory protein Bcl-2 significantly increased 6 h after *F. necrophorum* infection (**Figure 4B**) (*P* < 0.05). Two hours after *F. necrophorum* infection, the protein expressions of pro-apoptotic AIF (**Figure 4C**) and cyto-c (**Figure 4D**), the protein expression ratio of the cleaved-caspase-3/caspase-3 (**Figure 4E**), the protein expression ratio of the cleaved-caspase-9/caspase-9 (**Figure 4F**), and the protein expression ratio of the cleaved-caspase-8/caspase-8 (**Figure 4G**) were significantly increased (*P* < 0.05). At the same time, the protein expression levels of IL-6, TNF-α, and IL-1β protein were detected by Western blot and ELISA, the results showed that the expression of IL-6 (**Figures 4H, K, P**), TNF-α (**Figures 4I, L, O**), and IL-1β (**Figures 4J, M, N**) significantly increased after *F. necrophorum* infection (*P* < 0.01). The results showed that *F. necrophorum* promoted apoptosis and the expression of inflammatory factors. These results suggested that *F. necrophorum* promoted macrophage apoptosis.

### *F. necrophorum* Promoted Sheep Neutrophils to undergo Apoptosis and Inflammation

**Figure 5:**
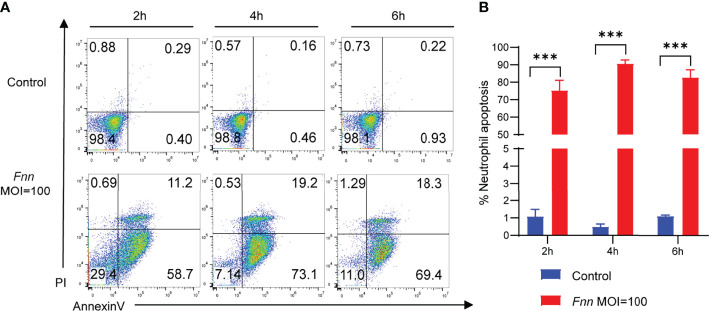
Apoptosis of sheep neutrophils cells induced by *F. necrophorum* (MOI=100). **(A)** Flow cytometry analysis of sheep neutrophil cells apoptosis with *F. necrophorum* stimulation (MOIs of 0 and 100) at 2, 4, and 6 h. **(B)** Statistical analysis of sheep neutrophils apoptosis with *F. necrophorum* stimulation (MOIs of 0 and 100) at 2, 4, and 6 h. (n = 3). ***P<0.001.

The Annexin V-FITC/PI method was used to detect the *F. necrophorum*−infected apoptotic rate of neutrophils with the MOI of 0 and 100:1 for 2, 4, and 6 h for investigating the *F. necrophorum*−induced apoptosis of neutrophils. Flow cytometry showed that the apoptotic rate of neutrophils in the control group was 0.69%, 0.62%, and 1.15%. The apoptotic rate of the experimental group was 58.7%, 92.3%, and 87.8%, respectively (**Figure 5A**), indicating that neutrophils were induced to undergo apoptosis by *F. necrophorum*. The statistical analysis of data revealed that the *F. necrophorum*−infected apoptotic rate of neutrophils differed significantly at different times compared with that in the control group (*P* < 0.01) (**Figure 5B**).

**Figure 6:**
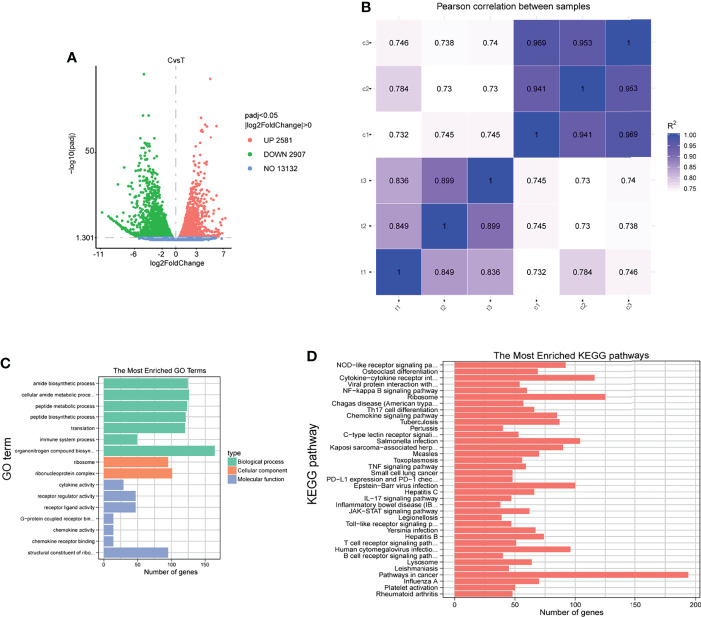
RNA-sequencing analysis of sheep neutrophils cells stimulated with *F. necrophorum* (MOI of 100). **(A)** The number of DEGs in six samples after *F. necrophorum* stimulation at 4 h. **(B)** Pearson correlation between samples. **(C)** GO enrichment analysis of the DEGs. **(D)** KEGG enrichment analysis of the DEGs. (*n* = 3).

We selected cells infected for 4 h to better understand the overall response of sheep neutrophils to *F. necrophorum* infection. We performed genome-wide transcriptional analysis using RNA-seq to determine the changes in gene expression. Further, 41-GB sequencing data were generated from six samples, with an average of 45.18 million reads per sample (**Table 3**). After QC, clean reads were mapped to reference genomes and transcripts, with mapping percentages of 63.46% and 87.42%, respectively (**Table 4**). A total of 28013 genes were detected, and the expression levels were calculated with FPKM. The gene expression profiles of normal sheep neutrophils were compared with those of infected cells after 4 h to characterize the DEGs influenced by *F. necrophorum*. Approximately 2581 genes were found to be upregulated, and 2907 genes were downregulated (**Figure 6A**). The correlation between samples is shown by Pearson correlation between samples (**Figure 6B**). Sixteen significant differences in GO function enrichment analysis were detected, including seven biological processes, two cell components, and seven molecular functions. After infection with *F. necrophorum*, the functions of sheep neutrophils mainly focused on the activities of cytokines, transcription, and ribosomes (**Figure 6C**; **Table S1**). The KEGG annotation showed that the top five enriched pathways were NOD (nucleotide binding oligomerization domain containing)-like receptor signaling pathway, osteoclast differentiation, cytokine-cytokine receptor interaction, viral protein interaction, and NF-κB signaling pathway (**Figure 6D**; **Table S2**).

**Table 3:** Transcriptome data QC results.

Sample	Raw-reads	Clean-reads	Error-rate
c1	45032186	43882714	0.02
c2	47293778	45768740	0.02
c3	47712492	45913948	0.02
t1	44488008	43349748	0.03
t2	48095788	46248220	0.03
t3	47470882	46207042	0.03

c1, c2, c3 representations the control group;t1, t2, and t3 represent the F. necrophorum-infected group.

**Table 4:** Transcriptome samples and reference genome comparison results.

Sample	Total-reads	Total-map
c1	43882714	38613441(87.99%)
c2	45768740	39367097(86.01%)
c3	45913948	40520129(88.25%)
t1	43349748	26794666(61.81%)
t2	46248220	29643382(64.1%)
t3	46207042	29788272(64.47%)

c1, c2, c3 representations the control group;t1, t2, and t3 represent the F. necrophorum-infected group.

**Figure 7:**
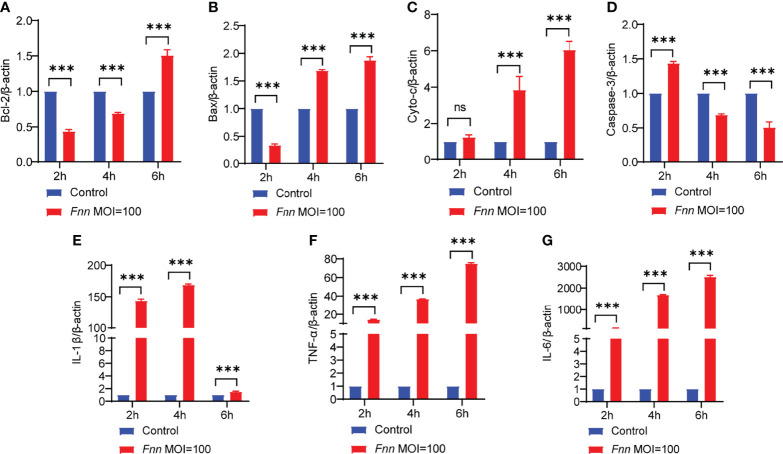
The effect of *F. necrophorum* on apoptosis and inflammatory factors of sheep neutrophils cells was detected by RT-qPCR. Bcl-2 **(A)**, Bax **(B)**, cyto-c **(C)**, caspase-3 **(D)**, IL-1β **(E)**, TNF-α **(F)** and IL-6 **(G)** gene expression with *F. necrophorum* stimulation (MOIs of 0 and 100) from 0 to 6 h (*n* = 3). ***P<0.001, ns, not significant.

The apoptosis-related genes Bcl-2, caspase-3, Bax, AIF, cyto-c, and inflammatory genes TNF-α, IL-1β, and IL-6 were detected by RT-qPCR to comprehend the changes in the *F. necrophorum*−induced apoptosis of sheep neutrophils. The results showed that the expression of pro-apoptotic genes Bax (**Figure 7B**), cyto-c (**Figure 7C**), and caspase-3 (**Figure 7D**) was upregulated, and the expression of anti-apoptotic gene Bcl-2 (**Figure 7A**) was downregulated (*P* < 0.01). Also, *F. necrophorum* promoted the apoptosis of sheep neutrophils at the gene level. The expression of pro-inflammatory factors IL-1β (**Figure 7E**), TNF-α (**Figure 7F**), and IL-6 (**Figure 7G**) was upregulated to varying degrees (*P <* 0.01). The results showed that the gene-level change was consistent with the transcriptomic results. Therefore, the *F. necrophorum*−induced apoptosis of sheep neutrophils was closely related to the occurrence of inflammation.

### *F. necrophorum* Induced Apoptosis and Inflammation through NF-κB and Death Receptor Signaling Pathways

**Figure 8:**
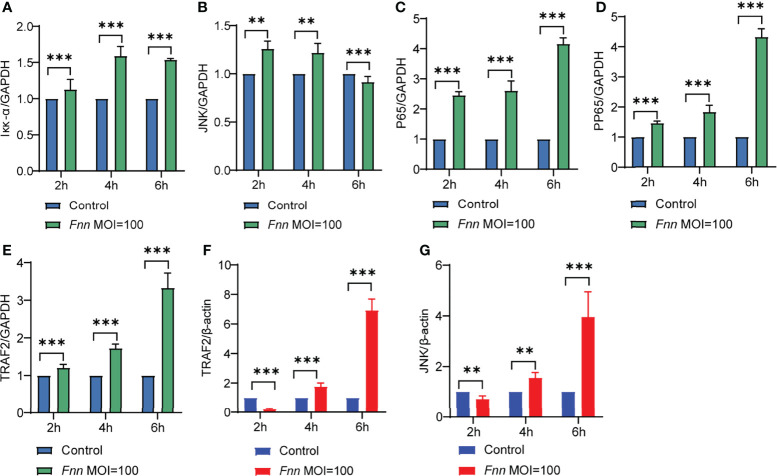
Effects of *F. necrophorum* the activation of the NF-κB and mitochondrial signaling pathways in cells. Iκκα **(A)**, JNK **(B)**, NF-κB p65 (p65) **(C)**, phosphorylated NF-κB p65(pp65) **(D)** and TRAF2 **(E)** gene expression with *F. necrophorum* stimulation macrophages (MOIs of 0 and 100) from 0 to 6 h (*n* = 3). TRAF2 **(F)** and JNK **(G)** gene expression with *F. necrophorum* stimulation sheep neutrophils cells (MOIs of 0 and 100) from 0 to 6 h (*n* = 3). **P<0.01, ***P<0.001.

The transcriptome results of *F. necrophorum*−infected sheep neutrophils integrated and analyzed through the pathway view. As shown in **Supplementary Figures 1**, **2**, DEGs were clustered in the NF-κB signaling pathway and the death receptor signaling pathway. RT-qPCR was used to detect the regulation of NF-κB and death receptor signaling pathways in *F. necrophorum*−infected neutrophils and macrophages. The expression levels of TRAF2, JNK, Iκκα, p65, and pp65 in macrophages and those of TRAF2, and JNK in sheep neutrophils were detected. The results showed that the relative mRNA expression levels of Iκκα (**Figure 8A**), JNK (**Figure 8B**), NF-κB p65 (p65) (**Figure 8C**), phosphorylated NF-κB p65 (pp65) (**Figure 8D**), and TRAF2 (**Figure 8E**) were upregulated in macrophages (*P <* 0.01), and the relative mRNAs expression levels of TRAF2 (**Figure 8F**) and JNK (**Figure 8G**) were upregulated in sheep neutrophils after *F. necrophorum* infection (*P <* 0.01).

**Figure 9:**
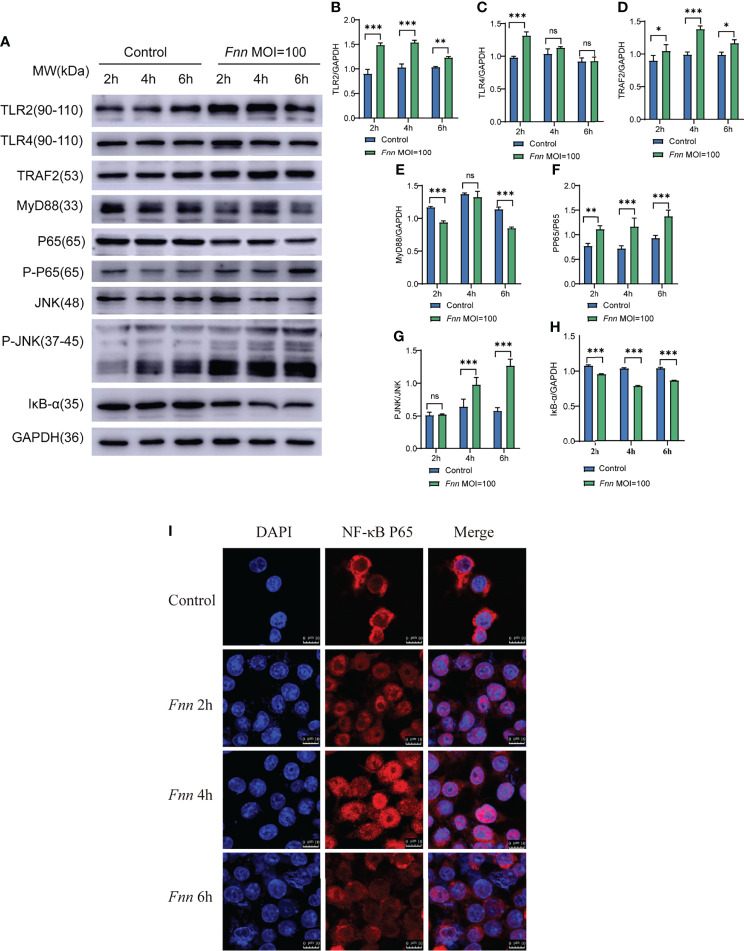
The effect of *F. necrophorum* on NF-κB and death receptor signaling pathways of macrophages was detected by Western blot and Immunofluorescence. **(A)** The protein levels of TLR2, TLR4, TRAF2, MyD88, NF-κB p65, phosphorylated NF-κB p65, JNK, phosphorylated JNK, and IκBα were detected by Western blot. TLR2 **(B)**, TLR4 **(C)**, TRAF2 **(D)**, MyD88 **(E)**, phosphorylated NF-κB p65/NF-κB p65 **(F)**, phosphorylated JNK/JNK **(G)**, and IκBα **(H)** was detected. Immunofluorescence images of NF-κB p65 in cells **(I)** (*n* = 3). *P<0.05, **P<0.01, ***P<0.001, ns, not significant.

**Figure 10:**
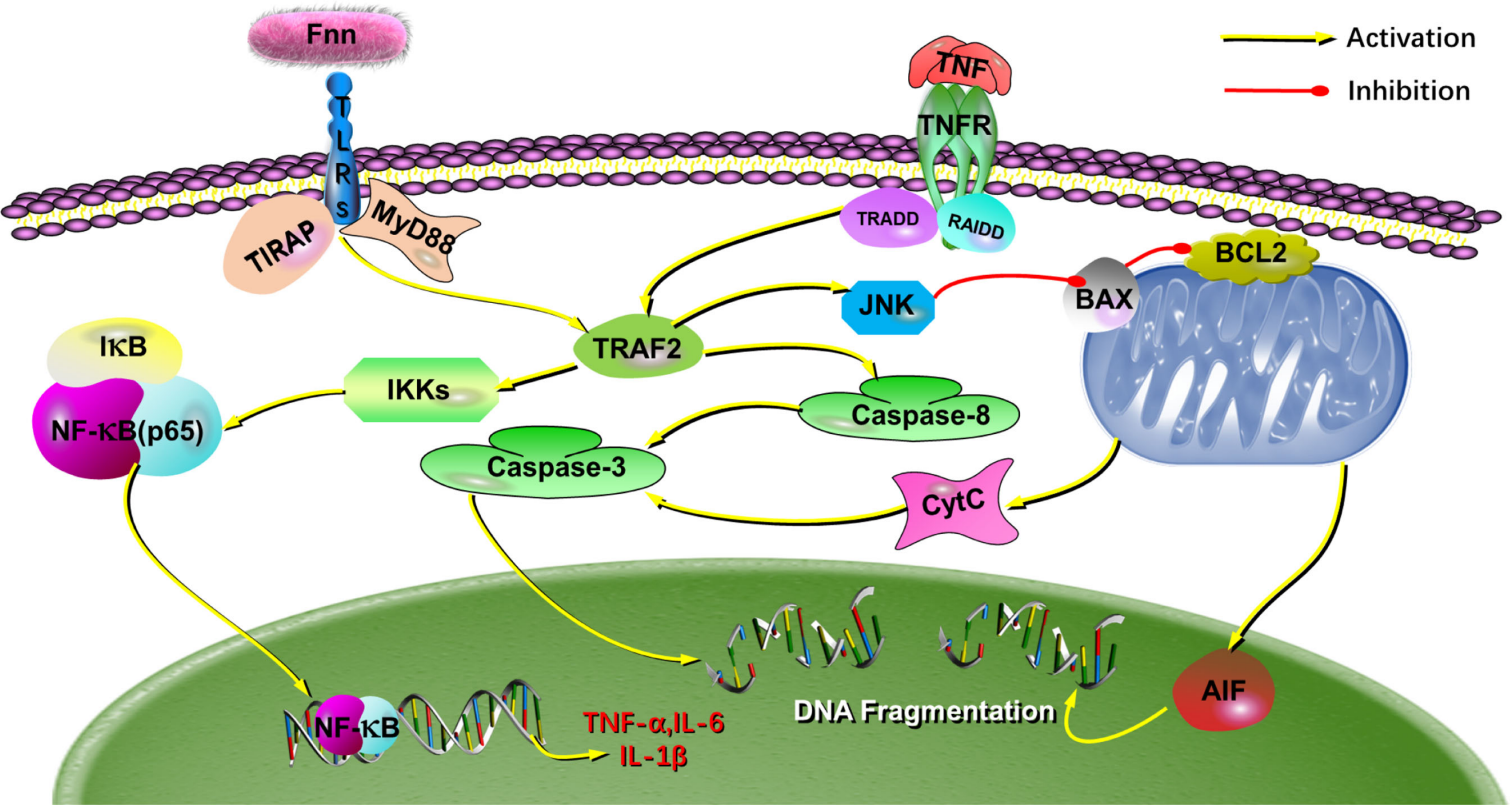
The putative mechanism for the effects of *F. necrophorum* on the biological processes in cells. The schematic diagram depicts how *F. necrophorum* activates NF-κB and death receptor signaling pathways, inducing cell apoptosis and inflammatory response. TLRs, Toll-like receptors; IκBα, inhibitors of NF-κB; NF-κB, nuclear factor kappa B.

Western blot results shown in **Figure 9A**. After infection with *F. necrophorum*, the protein expression of TLR2 (**Figure 9B**), TLR4 (**Figure 9C**), TRAF2 (**Figure 9D**), MyD88 (**Figure 9E**), the protein expression ratio of the pp65/p65 (**Figure 9F**), and the protein expression ratio of the p-JNK/JNK (**Figure 9G**) significantly upregulated (*P <* 0.05), the protein expression ratio of the IκB-α (**Figure 9H**) significantly downregulated (*P <* 0.05). Immunofluorescence cell analysis showed that the infection of *F. necrophorum* promoted the translocation of p65 from cytoplasm to nucleus (**Figure 9I**). The RT-qPCR and Western blot assay results were consistent with the transcriptomic results after immunization of *F. necrophorum*−infected cells. To summaries, when host cells are infected by *F. necrophorum*, the TRAF2 is activated by TLR receptors and transmits signals downstream, which activates Iκκα and promotes IκB-α degradation. NF-κB p65 protein is transferred from cytoplasm into the nucleus, resulting in the release of TNF-α, IL-6 and IL-1β. Meanwhile, during cell apoptosis, the death domain is activated first, TRAF2 is activated, and then caspase-8 is activated, cascade downstream caspase-3 to induce cell apoptosis. In addition, JNK phosphorylation is promoted, apoptosis-related proteins on mitochondria are regulated, and downstream caspase-3 is further regulated to induce apoptosis (**Figure 10**).

## Discussion

*F. necrophorum* is a critical pathogen isolated from oral cavities, gastrointestinal tracts, and genitourinary tracts of animals and humans (Tadepalli et al., 2009; Sato et al., 2021). It is frequently associated with necrotic infections in animals, such as calf diphtheria, foot rot, and liver abscesses (Langworth, 1977). The economic loss associated with foot rot and lameness in dairy and beef cattle and hepatic abscesses in feedlot cattle are of significant concern to the cattle industry. In feedlots, the incidence of liver abscesses averages 12%–32% depending on various management and dietary factors (Nagaraja and Chengappa, 1998; Nagaraja and Lechtenberg, 2007). In addition, *F. necrophorum* is also the causative agent of the invasive disease Lemierre′s syndrome and is associated with peritonsillar abscess formation and otitis media in children (Holm et al., 2016). These diseases tend to be necrotizing and abscessing in nature involving a large number of macrophages and neutrophils. Most research focused on adhesion effects (He et al., 2020), virulence factors (Pillai et al., 2019), and vaccine development (Xiao et al., 2021). Although it is known that *F. necrophorum* can induce apoptosis of macrophages and neutrophils (Narayanan et al., 2002), the underlying molecular mechanisms remain unclear. In this study, we found that *F. necrophorum* could induce the apoptosis of macrophages and neutrophils and product inflammatory factors *via* NF-κB and death receptor signaling pathways (**Figure 10**).

Apoptosis, a programmed cell death, triggered by an internally regulated suicidal program. A large number of pathogens can induce apoptosis in host cell and regulate the cellular pathway of inducing or inhibiting apoptosis (Wanford et al., 2022). These pathogens are significantly recognized by host proteins and stimulate a variety of signal pathways to change the stimulation, including phagocytosis, release of apoptotic and inflammatory cytokines, and the triggering of apoptosis (Selvaraj et al., 2021). *F. necrophorum* can not only inhibit macrophage proliferation, but also induced apoptosis of macrophages and sheep neutrophils, which is consistent with previous studies (Fales et al., 1977; Narayanan et al., 2002). Traditionally, the apoptosis of host cells can be divided into two pathways-extrinsic and intrinsic pathways (Galluzzi et al., 2018). Extrinsic pathway can be triggered by death receptors including Fas and tumor necrosis factor receptor (TNF-R), which can activate caspase-8, thus activating Caspase-3 and leading to apoptosis when adaptor proteins suffering from external stimulation (Yuan et al., 2018). Combined with the results of transcriptome, RT-qPCR and Western blot, the host cells infected with *F. necrophorum* will firstly active TRAF2, then promoted the expression of caspase-8 and caspse3, and finally caused apoptosis. Besides, the expression of pro-apoptotic genes (Bax, cyto-c, AIF, caspase-9, caspase-12) were significantly upregulated when cells infected with *F. necrophorum*.

In the present study, the transcriptome analysis of sheep neutrophils infected with *F. necrophorum* showed that these bacteria not only promoted apoptosis but also promoted the expression of pro-inflammatory genes. The production of inflammatory factors can provide a basis for the recreation of inflammatory cells during infection *in vivo* (Garcia et al., 2000). Previous transcriptome analysis has shown that rumen epithelial cells with and without a liver abscess has 221 DEGs mainly enriched in NF-κB and interferon signaling pathways (Abbas et al., 2020). *F. necrophorum* could also activate NF-κB signal path, which were obtained *via* transcriptome analysis in this research. The expression of TLRs could affect NF-κB pathway, and within the family of TLRs, TLR2 is considered as the main pattern recognizer of outer membrane protein in Gram-negative bacteria (Punturieri et al., 2006; Alva-Murillo et al., 2017). While TLR4 is considered as the main pattern recognition receptor of bacterial endotoxin. The activation of these two receptors lead to the release of inflammatory cytokines (Yang, 2022). It has been reported that the expression of these mediators is also regulated by NF-κB pathway (Alva-Murillo et al., 2017). NF-κB protein usually forms homologous/heterodimer from p65 and p50, and stay inactivated in the cytoplasm due to the combination with inhibitory protein IκBα, normally (Jiang et al., 2017). Once activated, NF-κB subunit p65 will isolated from the inhibitory protein IκBα, and moved to nucleus where it may trigger the transcription of specific target genes including TNF-α, IL-1β and IL-6. To further understanding the molecular mechanism of host cells in *F. necrophorum* infection, we examined the inflammatory cytokines (TNF-A, IL-1β, and IL-6), the key proteins in the NF-κB signaling pathway (Iκκα, IκBα), and the position transformation of NF-κB subunit P65. The results showed that *F. necrophorum* promoted the expression of inflammatory factors and TLRs (TLR2, TLR4), these findings are similar to those of previous studies whose transcriptome data were analyzed in sheep foot rot samples (Davenport et al., 2014). Besides, these bacteria significantly promoted phosphorylation of JNK and NF-κB, and also promoted the transformation of NF-κB subunit p65 from cytoplasm to nucleus. Therefore, it was concluded that the NF-κB and death receptor signaling pathways regulated *F. necrophorum*−induced cell apoptosis and inflammatory response.

In conclusion, our study showed that *F. necrophorum* inhibited the proliferation of immune cells, and promoted apoptosis and inflammatory cytokine production through the activation of NF-κB and death receptor signaling pathways. This is the first report in exploring the changes in *F. necrophorum*−infected apoptosis and expression of inflammatory factors in immune cells, which laid a foundation for investigating the infection mechanism of *F. necrophorum*.

## Data Availability Statement

The datasets presented in this study can be found in online repositories. The names of the repository/repositories and accession number(s) can be found below: https://www.ncbi.nlm.nih.gov/, PRJNA783192.

## Ethics Statement

The animal study was reviewed and approved by This study was approved by the Animal Health, Animal Care, and Use Committee of the Heilongjiang Bayi Agricultural University.

## Author Contributions

D-HG and D-BS designed the project and experiments. F-FW, T-SW and KJ conducted the experiments. X-JH and P-YZ analyzed the data. F-FW and J-WX made the images. F-FW and D-HG prepared the manuscript. All authors read and approved the final version of the manuscript.

## Funding

This work was supported by the Natural Science Foundation of Heilongjiang Province of China (Grant No. LH2021C070), National Funds for Supporting Reform and Development of Heilongjiang Provincial Colleges and Universities (Grant No. 2022010009), and the National Natural Science Foundation of China (Beijing, China; Grant No. 31572534).

## Conflict of Interest

The authors declare that the research was conducted in the absence of any commercial or financial relationships that could be construed as a potential conflict of interest.

## Publisher’s Note

All claims expressed in this article are solely those of the authors and do not necessarily represent those of their affiliated organizations, or those of the publisher, the editors and the reviewers. Any product that may be evaluated in this article, or claim that may be made by its manufacturer, is not guaranteed or endorsed by the publisher.

## Supplementary Material

Supplementary Figure 1 The Pathview analysis of the NF-κB signaling pathway. The gene expression level is calculated by the log fold change in the *F. necrophorum* stimulation group relative to the control group.

Supplementary Figure 2 The Pathview analysis of the apoptosis signaling pathway. The gene expression level is calculated by the log fold change in the *F. necrophorum* stimulation group relative to the control group.
