# Supplementary figures and images for: Fusobacterium necrophorum Promotes Apoptosis and Inflammatory Cytokine Production Through the Activation of NF-κB and Death Receptor Signaling Pathways
Source: Front Cell Infect Microbiol. 2022 Jun 14;12:827750. doi: 10.3389/fcimb.2022.827750 (PMC9237437; doi:10.3389/fcimb.2022.827750)

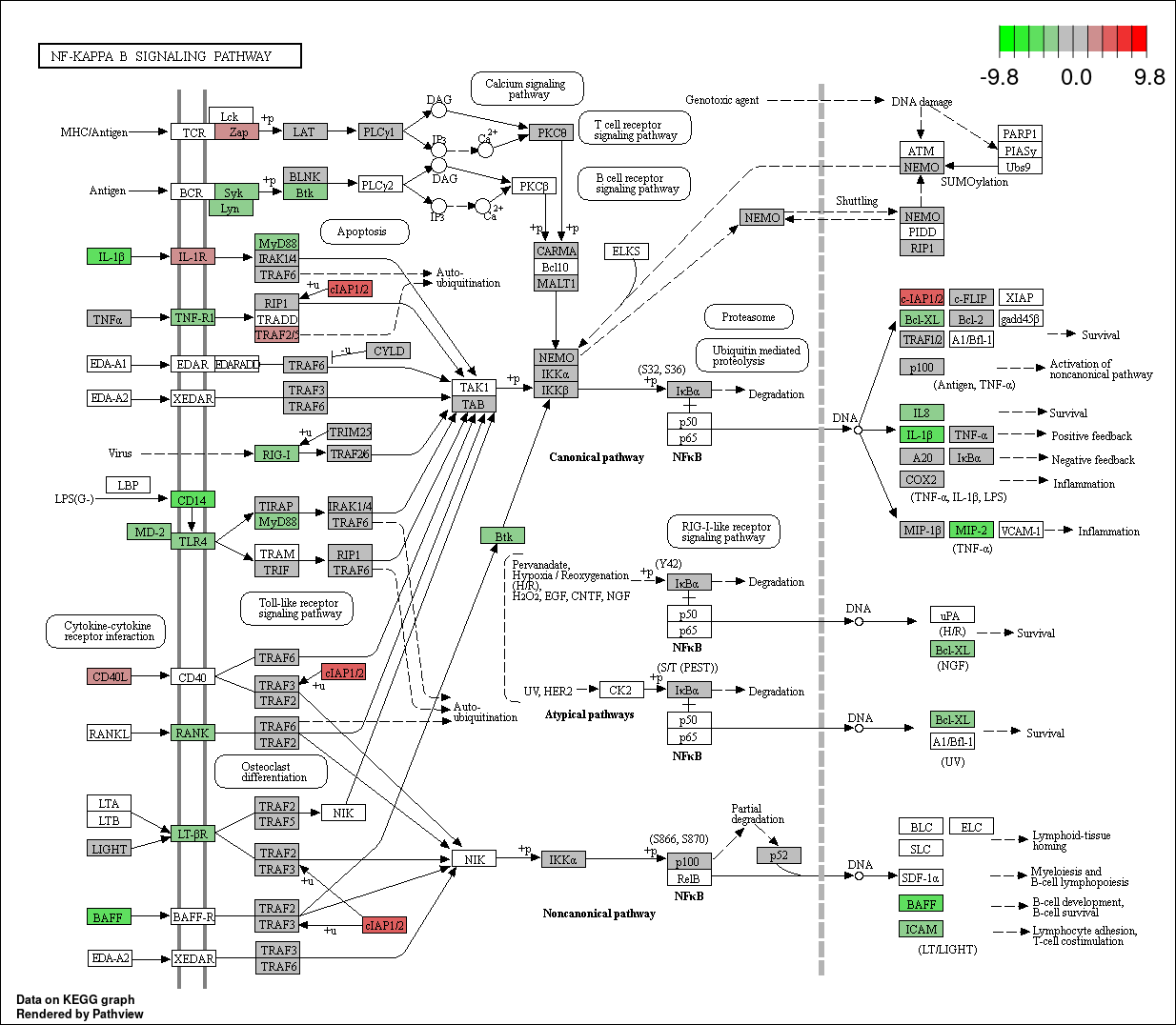

Supplement: Supplementary Figure 1 — The Pathview analysis of the NF-κB signaling pathway. The gene expression level is calculated by the log fold change in the F. necrophorum stimulation group relative to the control group. [file Image_1.png]

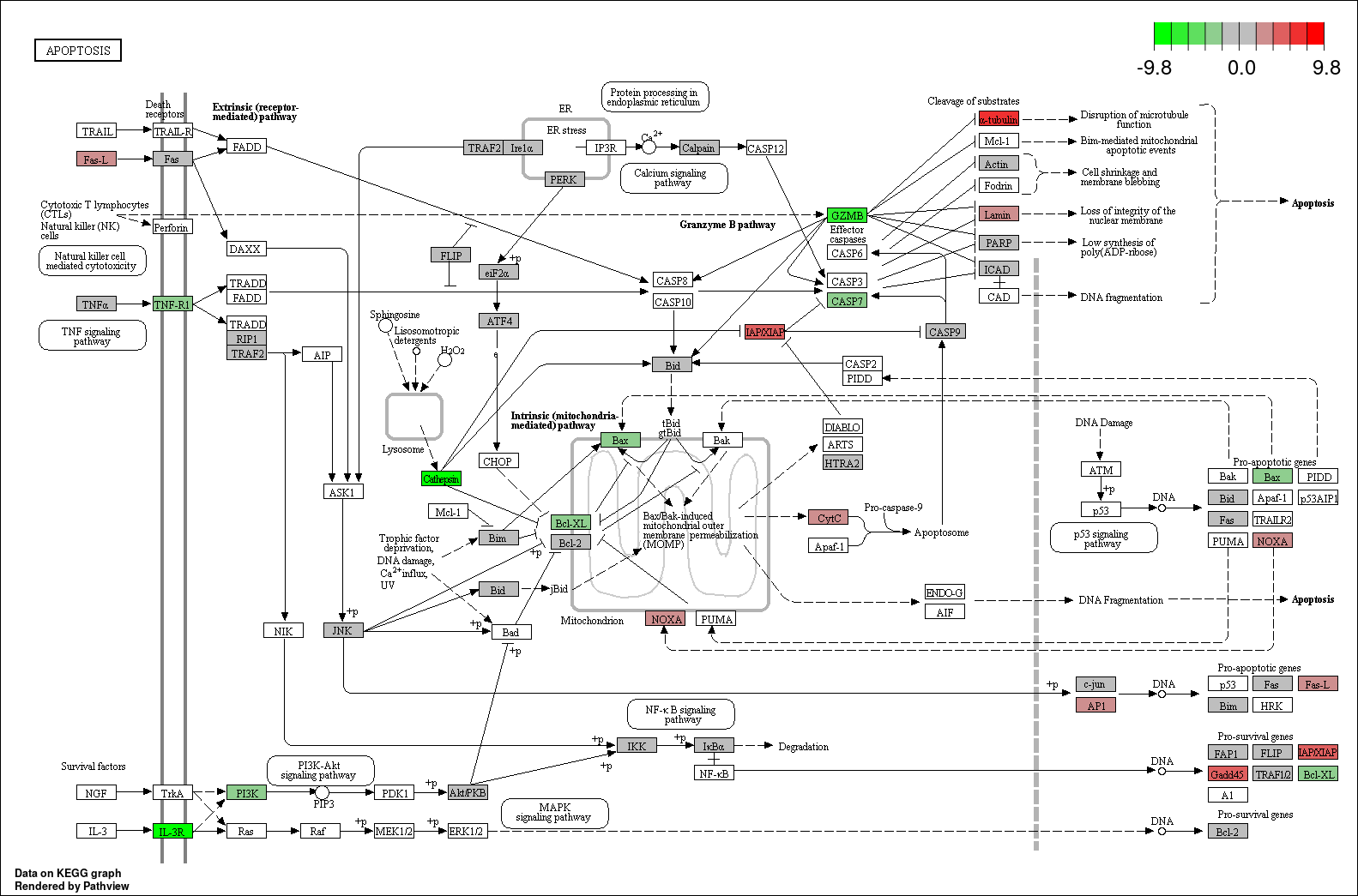

Supplement: Supplementary Figure 2 — The Pathview analysis of the apoptosis signaling pathway. The gene expression level is calculated by the log fold change in the F. necrophorum stimulation group relative to the control group. [file Image_2.png]
