# Supplementary material for: Fusobacterium necrophorum Promotes Apoptosis and Inflammatory Cytokine Production Through the Activation of NF-κB and Death Receptor Signaling Pathways
Source: Front Cell Infect Microbiol. 2022 Jun 14;12:827750. doi: 10.3389/fcimb.2022.827750 (PMC9237437; doi:10.3389/fcimb.2022.827750)
Supplement: Supplementary file 3 [file Table_1.docx]

**Supplementary Table 1:** Statistical table of GO enrichment analysis of differential genes

| Category | GO ID | Description | pvalue | padj |
| --- | --- | --- | --- | --- |
| Biological process | GO:0043604 | amide biosynthetic process | 0.000104 | 0.021951 |
| Biological process | GO:0043603 | cellular amide metabolic process | 0.000117 | 0.021951 |
| Biological process | GO:0006518 | peptide metabolic process | 0.000169 | 0.021951 |
| Biological process | GO:0043043 | peptide biosynthetic process | 0.000215 | 0.021951 |
| Biological process | GO:0006412 | translation | 0.000243 | 0.021951 |
| Biological process | GO:0002376 | immune system process | 0.000484 | 0.036371 |
| Biological process | GO:1901566 | organonitrogen compound biosynthetic process | 0.000588 | 0.037886 |
| Cellular component | GO:0005840 | ribosome | 9.97E-05 | 0.012061 |
| Cellular component | GO:1990904 | ribonucleoprotein complex | 0.000343 | 0.020779 |
| Molecular function | GO:0005125 | cytokine activity | 1.20E-06 | 0.000326 |
| Molecular function | GO:0030545 | receptor regulator activity | 0.000281 | 0.025604 |
| Molecular function | GO:0048018 | receptor ligand activity | 0.000281 | 0.025604 |
| Molecular function | GO:0001664 | G-protein coupled receptor binding | 0.000632 | 0.028763 |
| Molecular function | GO:0008009 | chemokine activity | 0.000632 | 0.028763 |
| Molecular function | GO:0042379 | chemokine receptor binding | 0.000632 | 0.028763 |
| Molecular function | GO:0003735 | structural constituent of ribosome | 0.000782 | 0.030482 |
