# Supplementary material for: Fusobacterium necrophorum Promotes Apoptosis and Inflammatory Cytokine Production Through the Activation of NF-κB and Death Receptor Signaling Pathways
Source: Front Cell Infect Microbiol. 2022 Jun 14;12:827750. doi: 10.3389/fcimb.2022.827750 (PMC9237437; doi:10.3389/fcimb.2022.827750)
Supplement: Supplementary file 4 [file Table_2.docx]

| KEGGID | Description | pvalue | padj |
| --- | --- | --- | --- |
| oas04621 | NOD-like receptor signaling pathway | 6.80E-09 | 2.15E-06 |
| oas04380 | Osteoclast differentiation | 1.64E-08 | 2.18E-06 |
| oas04060 | Cytokine-cytokine receptor interaction | 2.07E-08 | 2.18E-06 |
| oas04061 | Viral protein interaction with cytokine and cytokine receptor | 4.21E-08 | 3.32E-06 |
| oas04064 | NF-kappa B signaling pathway | 3.33E-06 | 0.000185 |
| oas03010 | Ribosome | 3.51E-06 | 0.000185 |
| oas05142 | Chagas disease (American trypanosomiasis) | 1.46E-05 | 0.000661 |
| oas04659 | Th17 cell differentiation | 2.11E-05 | 0.000793 |
| oas04062 | Chemokine signaling pathway | 2.44E-05 | 0.000793 |
| oas05152 | Tuberculosis | 2.70E-05 | 0.000793 |
| oas05133 | Pertussis | 3.01E-05 | 0.000793 |
| oas04625 | C-type lectin receptor signaling pathway | 3.01E-05 | 0.000793 |
| oas05132 | Salmonella infection | 8.87E-05 | 0.002142 |
| oas05167 | Kaposi sarcoma-associated herpesvirus infection | 0.000101 | 0.002142 |
| oas05162 | Measles | 0.000102 | 0.002142 |
| oas05145 | Toxoplasmosis | 0.000204 | 0.003525 |
| oas04668 | TNF signaling pathway | 0.000212 | 0.003525 |
| oas05222 | Small cell lung cancer | 0.000212 | 0.003525 |
| oas05235 | PD-L1 expression and PD-1 checkpoint pathway in cancer | 0.000212 | 0.003525 |
| oas05169 | Epstein-Barr virus infection | 0.000314 | 0.004965 |
| oas05160 | Hepatitis C | 0.000352 | 0.0053 |
| oas04657 | IL-17 signaling pathway | 0.000468 | 0.006721 |
| oas05321 | Inflammatory bowel disease (IBD) | 0.000501 | 0.00681 |
| oas04630 | JAK-STAT signaling pathway | 0.000517 | 0.00681 |
| oas05134 | Legionellosis | 0.000674 | 0.008525 |
| oas04620 | Toll-like receptor signaling pathway | 0.0009 | 0.010665 |
| oas05135 | Yersinia infection | 0.000911 | 0.010665 |
| oas05161 | Hepatitis B | 0.000957 | 0.010802 |
| oas04660 | T cell receptor signaling pathway | 0.001494 | 0.015829 |
| oas05163 | Human cytomegalovirus infection | 0.001503 | 0.015829 |
| oas04662 | B cell receptor signaling pathway | 0.001748 | 0.017815 |
| oas04142 | Lysosome | 0.002472 | 0.024413 |
| oas05140 | Leishmaniasis | 0.002674 | 0.025608 |
| oas05200 | Pathways in cancer | 0.003043 | 0.02828 |
| oas05164 | Influenza A | 0.003339 | 0.03015 |
| oas04611 | Platelet activation | 0.004686 | 0.041137 |
| oas05323 | Rheumatoid arthritis | 0.00569 | 0.048599 |

**Supplementary Table 2:** Statistical table of KEGG enrichment analysis of differential genes
